# Visceral adiposity index (VAI), lipid accumulation product (LAP), and product of triglycerides and glucose (TyG) to discriminate prediabetes and diabetes

**DOI:** 10.1038/s41598-019-46187-8

**Published:** 2019-07-04

**Authors:** Nayeon Ahn, Sebastian E. Baumeister, Ute Amann, Wolfgang Rathmann, Annette Peters, Cornelia Huth, Barbara Thorand, Christa Meisinger

**Affiliations:** 1 Chair of Epidemiology, Ludwig-Maximilians-Universität München, UNIKA-T Augsburg, Augsburg, Germany; 2 The Institute of Medical Informatics, Biometry and Epidemiology (IBE), Ludwig-Maximilians-University of Munich, Munich, Germany; 3 Institute for Biometrics and Epidemiology, German Diabetes Center, Leibniz Center for Diabetes Research at Heinrich Heine University, Düsseldorf, Germany; 4 Institute of Epidemiology, Helmholtz Zentrum München, German Research Center for Environmental Health, Neuherberg, Germany; 5 Independent Research Group Clinical Epidemiology, Helmholtz Zentrum München, German Research Center for Environmental Health, Neuherberg, Germany

**Keywords:** Diagnostic markers, Body mass index

## Abstract

The present study evaluated the ability of the visceral adiposity index (VAI), the lipid accumulation product (LAP), and product of triglycerides and glucose (TyG), three novel, insulin resistance-related markers, to discriminate prediabetes/diabetes in the general German population. Altogether 2,045 Germans (31–72 years, 53.3% women) without known diabetes and a history of Myocardial Infarction (MI)/stroke from the Cooperative Health Research in the Region of Augsburg (KORA) F4 Study were eligible. The discriminatory accuracy of the markers for oral glucose tolerance test (OGTT)-defined prediabetes/diabetes according to the American Diabetes Association (ADA) criteria was assessed by the area under the receiver operating characteristic (ROC) curve (AUC). The Youden Index (YI) was used to determine optimal cut-off values, and a non-parametric ROC regression was used to examine whether the discriminatory accuracy varied by sex and age. 365 men (38.2%) and 257 women (23.6%) were newly diagnosed with prediabetes/diabetes. AUCs for TyG, LAP and VAI were 0.762 (95% CI 0.740–0.784), 0.743 (95% CI 0.720–0.765), and 0.687 (95% CI 0.662–0.712), respectively. The optimal cut-off values for the LAP and TyG were 56.70 and 8.75 in men, and 30.40 and 8.53 in women. In conclusion, TyG and LAP provide good discrimination of persons with prediabetes/diabetes.

## Introduction

The incidence and prevalence of type 2 diabetes (hereafter, diabetes) are increasing rapidly worldwide due to aging of the population and the Western lifestyle^1,2^. According to diabetes burden research by the International Diabetes Federation (IDF), it was estimated that there were 451 million adults with diabetes worldwide in 2017, and it is predicted that 693 million adults will have diabetes by 2045^3^. A large proportion of all people living with diabetes (IDF estimates 49.7%) does not know that they have the disease. Although timely treatment of diabetes reduces the risk of diabetes-related outcomes^4^, this requires efficient screening strategies and early detection of prediabetes and diabetes. The oral glucose tolerance test (OGTT) is currently the gold standard for the diagnosis of prediabetes/diabetes, but it is costly and inconvenient. Therefore, the development of accurate and cost-efficient screening tools as an alternative is warranted.

Insulin resistance (IR) is a metabolic state where insulin-dependent tissues become less sensitive to the actions of insulin, leading to an imbalance in metabolism. The pathogenic association of IR with prediabetes/diabetes, as well as with cardiovascular disease (CVD) is well recognized^5,6^.

Homeostatic model assessment of insulin resistance (HOMA-IR) has been used as a robust surrogate marker for defining IR because of the practical, ethical and economic issues of the hyperinsulinemic euglycemic clamp, which is the gold standard test for measurement of IR^7^. However, for the calculation of the HOMA-IR, the measurement of fasting serum glucose (FSG) (mg/dl) and fasting insulin (μu/ml) is necessary^7^. In recent years, several studies have suggested that novel indices, such as the visceral adiposity index (VAI), a model computed by both anthropometric (body mass index [BMI] and waist circumference [WC]) and laboratory (triglycerides [TG] and high-density lipoprotein cholesterol [HDL-C]) parameters, and the lipid accumulation product (LAP), a model based on a combination of TG and WC, might be accurate markers of IR^8–10^. Likewise, TyG, the product of TG (mg/dl) and FSG (mg/dl), has shown high accuracy for IR^11,12^.

Given that IR is the critical pathophysiologic mechanism of diabetes and is already present ahead of the diagnosis, the novel surrogate indices of IR might be useful in the identification of prediabetes/diabetes^5,6^.

To the best of our knowledge, limited evidence is available that provides data regarding the discriminatory accuracy of the three previously mentioned novel lipid indices for detecting prediabetes/diabetes. Additionally, previous reports have not tested whether discriminatory accuracy varies by sex and age^13,14^.

In this study, we set our sights on determining whether the VAI, LAP, and TyG could be valuable markers for detecting prediabetes/diabetes in a European population for the first time, while also conducting the first comparison of these three novel markers and HOMA-IR. We also aimed to define sex- and age-specific cut-off values.

## Results

### Baseline clinical characteristics and laboratory results

Table 1 provides an overview of the anthropometric and clinical characteristics of the study subjects. Of 2,045 subjects, 955 (46.7%) were men and 1,090 (53.3%) were women. In the overall study population, 30.4% had prediabetes/newly diagnosed diabetes (38.2% in men vs. 23.6% in women). Age, BMI, WC, waist-to-hip ratio, FSG, serum glucose 2 hours post OGTT (2h-serum glucose), TG, Hemoglobin A1C (HbA1c), use of statins, hypertension, parents’ history of diabetes, HOMA-IR, VAI, LAP, and TyG were higher in the group of prediabetes/newly diagnosed diabetes compared to persons without diabetes. HDL-C and the number of current smokers were lower in prediabetic/diabetic than in non-diabetic subjects, regardless of sex. However, no significant difference was found in Homeostatic model assessment beta-cell function (HOMA-beta) between the two groups.

**Table 1:** Comparison of anthropometric and clinical characteristics of subjects: distribution by sex.

Variables	Men	Women	P-value
	(n = 955)	(n = 1,090)	
Prediabetes/Diabetes, n(%)	365 (38.2)	257 (23.6)	<0.001
Age (years)	51.5 ± 10.9	51.7 ± 11.0	0.677
BMI (kg/m²)	27.5 ± 4.0	26.6 ± 5.1	<0.001
Waist Circumference (cm)	98.2 ± 11.9	86.3 ± 12.8	<0.001
Waist-Hip ratio (%)	92.5 ± 6.5	81.5 ± 6.8	<0.001
Fasting serum glucose (mg/dl)	97.5 ± 13.6	91.5 ± 10.6	<0.001
Fasting serum glucose (mmol/l)	5.9 ± 0.8	5.5 ± 0.8	
2h-serum glucose (mg/dl)	109.1 ± 39.7	107.3 ± 35.3	0.273
2h-serum glucose (mmol/l)	6.5 ± 2.4	6.4 ± 2.1	
TC (mmol/l)	5.60 ± 1.00	5.58 ± 1.03	0.665
TG (mmol/l)	1.69 ± 1.06	1.27 ± 0.65	<0.001
HDL-C (mmol/l)	1.30 ± 0.32	1.57 ± 0.35	<0.001
HbA1c (%)	5.41 ± 0.47	5.36 ± 0.35	0.019
Current smoking, n (%)	224 (23.5)	191 (17.5)	0.001
Alcohol consumption (g/day)	17.14 (2.9–22.4)	2.86 (0.0–11.7)	<0.001
Physical activity, n (%)	553 (57.9)	639 (58.6)	0.777
Taking statins, n (%)	66 (6.9)	63 (5.7)	<0.001
Hypertension, n (%)	312 (32.7)	259 (23.8)	<0.001
Parents’ history of DM, n(%)	215 (22.5)	276 (25.3)	0.258
: do not know	158 (16.5)	156 (14.3)	
HOMA-beta	99.1 (73.1–140.3)	106.8 (81.3–144.1)	<0.001
HOMA-IR	2.09 (1.49–3.11)	1.75 (1.23–2.55)	<0.001
VAI	1.54 (0.99–2.51)	1.31 (0.91–2.00)	<0.001
LAP	46.4 (28.3–73.8)	29.3 (17.1–51.0)	<0.001
TyG	8.75 ± 0.55	8.43 ± 0.48	<0.001

Results are expressed as mean ± standard deviation or median (25th–75th percentile) for continuous variables. The number of subjects and percentage is presented for categorical variables. 2 h-serum glucose, serum glucose after 2 hours from OGTT; HOMA-IR, homeostasis model assessment of insulin resistance; VAI, visceral adiposity index; LAP, lipid accumulation product; TyG, the product of triglyceride and glucose; HOMA-beta, Homeostatic model assessment beta-cell function (%) = (20 × fasting serum insulin)/(fasting serum glucose − 3.5)^7^.

We found several differences between the sexes. There was no significant increase in statin use in prediabetic/diabetic male subjects compared to subjects without diabetes, yet higher use of statins was observed in prediabetic/diabetic female subjects compared to the non-diabetic females (10.1% vs. 4.4%, p = 0.001). Concerning alcohol consumption, prediabetics/diabetics men drank more alcohol than non-diabetic men did (20.0 g/day vs. 14.3 g/day, p < 0.001). While less physically active individuals were found more often in non-diabetic cases than persons with prediabetes/diabetes (60.0% vs. 54.3%, p = 0.019), no difference was observed when stratified by sex (men: 59.7% vs. 55.1% [p = 0.184], women: 60.3% vs. 53.3% [p = 0.057]).

### The discriminatory accuracy of VAI, LAP, and TyG for prediabetes/diabetes

**Figure 1:**
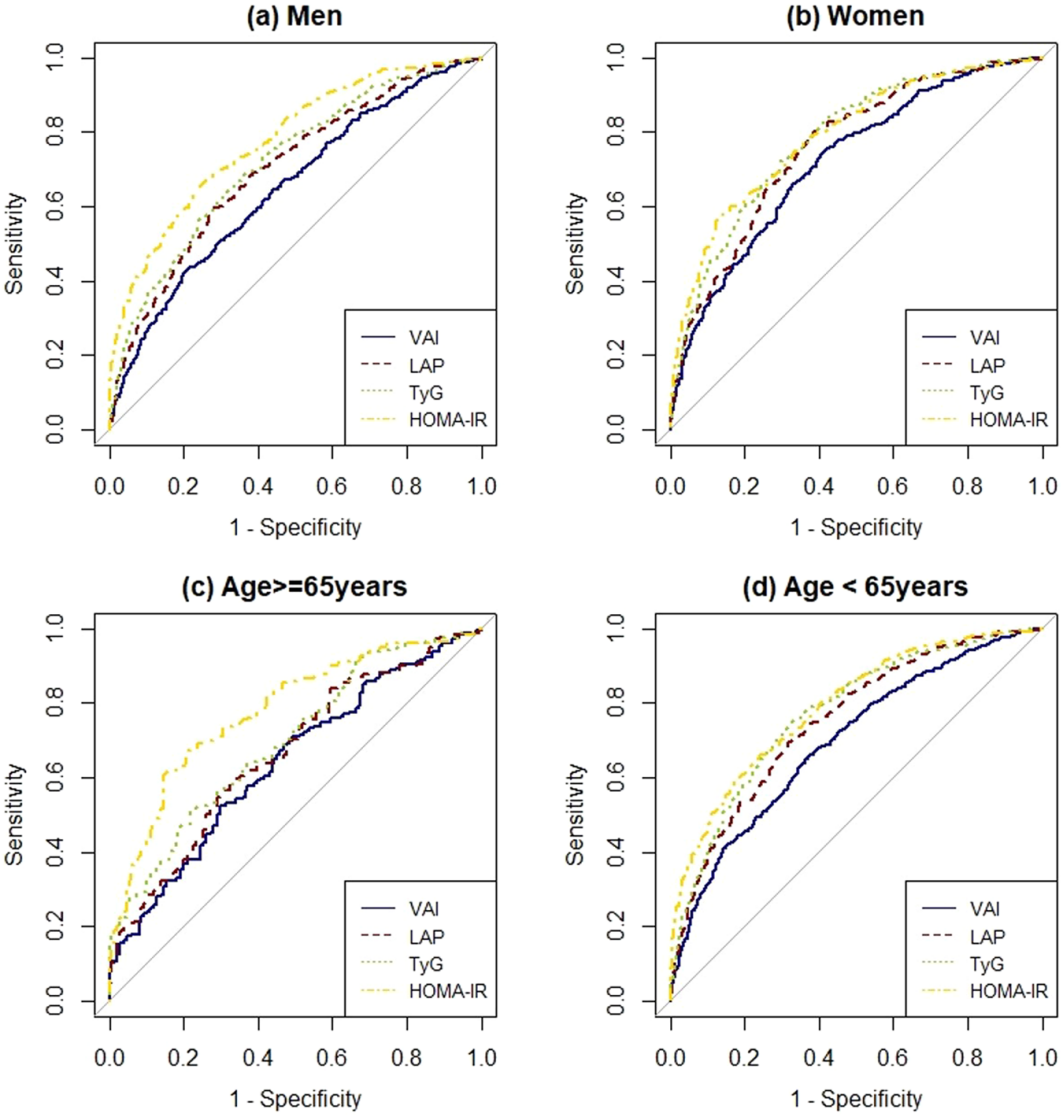
Comparison of the diagnostic value of visceral adiposity index (VAI), lipid accumulation product (LAP), the product of triglycerides and glucose (TyG), and homeostasis model assessment of insulin resistance (HOMA-IR) in (**a**) Men, (**b**) Women, (**c**) Age ≥ 65 years, and (**d**) Age < 65 years.

Measures of discriminatory accuracy are provided in Table 2 and Fig. 1. As expected, HOMA-IR had the highest AUC, followed by TyG and LAP in the overall study population. TyG showed the highest sensitivity (0.732) and NPV (0.849). The highest specificity (0.811) and PPV (0.589) were observed for HOMA-IR. The optimal cut-off values were 1.52 for VAI, 41.30 for LAP, and 8.60 for TyG. Regarding DLR, HOMA-IR had the highest DLR+ (3.283) followed by TyG (DLR+: 2.142), where DLR+ > 1 indicates increased probability of disease.

**Table 2:** Discriminatory accuracy and cut-off values for VAI, LAP, TyG, and HOMA-IR.

	Sensitivity	Specificity	PPV	NPV	AUC (95% CI)	P-value^†^	DLR(+)	DLR(−)	YI	Cut-offs	CV AUC (95% CI)
**Overall**											
VAI	0.645	0.629	0.432	0.802	0.687 (0.662–0.712)	<0.001	1.738	0.565	0.274	1.52	0.686 (0.661–0.711)
LAP	0.698	0.663	0.475	0.834	0.743 (0.720–0.765)	<0.001	2.073	0.456	0.361	41.30	0.742 (0.719–0.765)
TyG	0.732	0.658	0.484	0.849	0.762 (0.740–0.784)	0.015	2.142	0.408	0.390	8.60	0.761 (0.738–0.783)
HOMA-IR	0.621	0.811	0.589	0.830	0.790 (0.769–0.812)	NA	3.283	0.468	0.432	2.46	0.791 (0.769–0.812)
**Men**											
VAI	0.433	0.792	0.562	0.693	0.649 (0.613–0.685)	<0.001	2.076	0.716	0.224	2.33	0.648 (0.612–0.684)
LAP	0.595	0.727	0.574	0.744	0.701 (0.667–0.735)	<0.001	2.179	0.558	0.322	56.70	0.699 (0.665–0.733)
TyG	0.666	0.663	0.550	0.762	0.721 (0.687–0.754)	<0.001	1.974	0.504	0.328	8.75	0.717 (0.684–0.750)
HOMA-IR	0.671	0.741	0.616	0.785	0.779 (0.749–0.809)	NA	2.588	0.444	0.412	2.36	0.775 (0.745–0.806)
**Women**											
VAI	0.755	0.582	0.358	0.885	0.720 (0.685–0.755)	<0.001	1.807	0.421	0.337	1.32	0.720 (0.686–0.755)
LAP	0.802	0.613	0.390	0.909	0.760 (0.728–0.793)	0.052	2.074	0.323	0.415	30.40	0.761 (0.729–0.793)
TyG	0.720	0.705	0.429	0.891	0.782 (0.751–0.814)	0.672	2.438	0.398	0.425	8.53	0.783 (0.752–0.814)
HOMA-IR	0.584	0.857	0.558	0.870	0.790 (0.758–0.822)	NA	4.086	0.486	0.441	2.57	0.791 (0.760–0.822)
**Age** ≥ **65**											
VAI	0.525	0.702	0.676	0.555	0.639 (0.580–0.699)	<0.001	1.762	0.676	0.227	1.87	0.639 (0.580–0.698)
LAP	0.592	0.675	0.684	0.583	0.665 (0.607–0.723)	<0.001	1.825	0.604	0.268	49.95	0.678 (0.619–0.736)
TyG	0.508	0.781	0.734	0.573	0.697 (0.641–0.753)	0.005	2.326	0.629	0.290	8.85	0.700 (0.644–0.756)
HOMA-IR	0.609	0.854	0.832	0.648	0.779 (0.730–0.829)	NA	4.180	0.458	0.463	2.72	0.781 (0.732–0.830)
**Age** < **65**											
VAI	0.650	0.637	0.384	0.839	0.695 (0.667–0.723)	<0.001	1.790	0.549	0.287	1.52	0.694 (0.666–0.723)
LAP	0.695	0.682	0.432	0.865	0.750 (0.724–0.776)	<0.001	2.184	0.447	0.377	41.30	0.750 (0.725–0.776)
TyG	0.752	0.671	0.443	0.886	0.772 (0.747–0.797)	0.186	2.287	0.370	0.423	8.60	0.772 (0.747–0.797)
HOMA-IR	0.661	0.760	0.490	0.866	0.790 (0.766–0.814)	NA	2.758	0.445	0.422	2.27	0.790 (0.766–0.814)

VAI, visceral adiposity index; LAP, lipid accumulation product; TyG, the product of triglyceride and glucos; PPV, positive predictive value; NPV, negative predictive value; DLR, Diagnostic Likelihood Ratio; YI, Youden Index; CV AUC, 10-fold cross-validated AUC.

^†^P-value of receiver operating characteristic (ROC) test between each marker and benchmark (HOMA-IR), respectively.

In analyses stratified by sex and age, TyG showed higher diagnostic values for prediabetes/diabetes than VAI and LAP (Supplemental Table S1). When it comes to AUC comparisons using HOMA-IR as a benchmark, HOMA-IR showed a higher AUC than TyG in men (0.779 [0.749–0.809] vs. 0.721 [0.687–0.754]), p < 0.001) and in participants ≥65 years old (0.779 [0.730–0.829] vs. 0.697 [0.641–0.753], p = 0.005). On the contrary, no difference in AUCs between HOMA-IR and TyG was observed in women (0.790 [0.758–0.822] vs. 0.782 [0.751–0.814], p = 0.672) and in study participants younger than 65 years (0.790 [0.766–0.814] vs. 0.772 [0.747–0.797], p = 0.186). VAI and LAP behaved similarly across the subgroups. The best diagnostic performance of VAI was found in women (0.720 [0.685–0.755]) followed by participants younger than 65 years (0.695 [0.667–0.723]). The greatest AUC of LAP was detected in women (0.760 [0.728–0.793]), followed by accuracy in the group ‘age <65 years’ (0.750 [0.724–0.776]). There was no remarkable difference between the AUC and cross-validated AUC, which showed only a 0.000–0.013 difference of AUC regardless of the type of index, the sex and the age group.

### Sex- and age-specific non-parametric ROC regression analyses

Based on the primary results, a question of interest arose to determine how well the three lipid indices discriminate prediabetes/diabetes at different ages. Firstly, the variation of AUC by age was analysed using the direct ROC regression. AUCs of the three markers by sex and age are presented in Supplemental Table S2. Meanwhile, Supplemental Fig. S3 shows AUCs by age, along with corresponding 95% pointwise bootstrap confidence bands. The coefficient estimates for age were negative in the analysis stratified by sex, which suggested that the younger the person was at the time of examination, the better the discrimination of prediabetes/diabetes was expected to be shown. However, that effect was not significant for all of the three lipid indices. On the contrary, a significant negative linear effect of age on the accuracy of LAP and TyG for diagnosing prediabetes/diabetes was found in the overall population. Supplemental Table S2 provides the estimated AUC values at age 40, 55 and 70 years in the study population. Despite its downward trend in age, however, the confidence interval for the AUC of each marker was still higher than 0.5 at 70 years.

**Figure 2:**
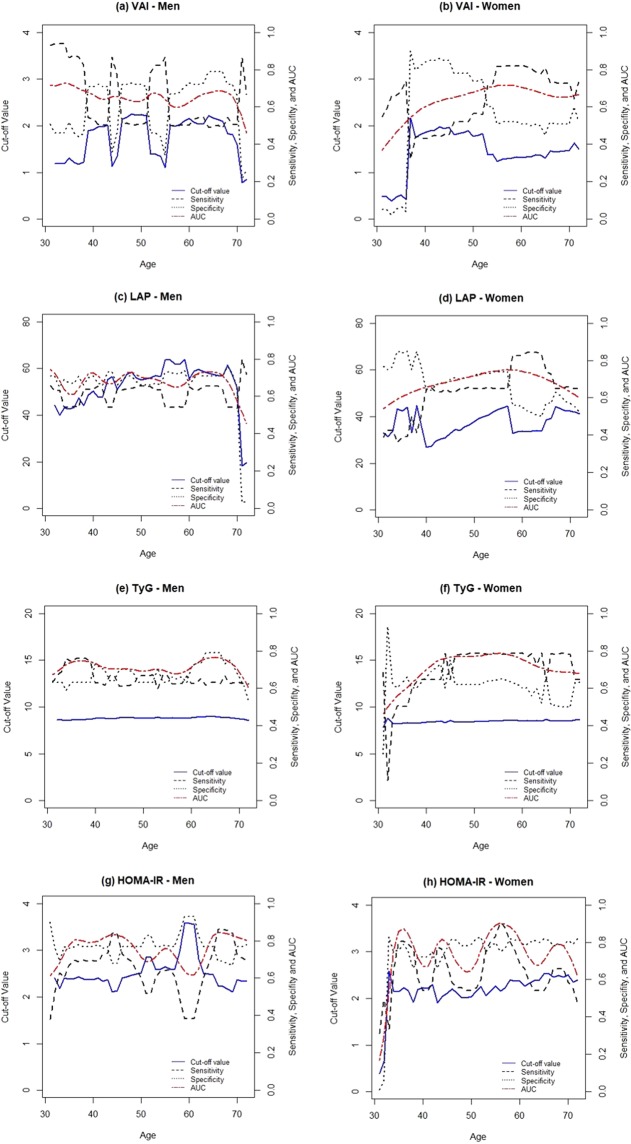
Optimal cut-off value, sensitivity, specificity, and AUC by age in men and women. (1000 Bootstrap replication). (**a**) VAI: men, (**b**) VAI: women, (**c**) LAP: men, (**d**) LAP: women, (**e**) TyG: men, (**f**) TyG: women, (**g**) HOMA: men, (**h**) HOMA-IR: women.

Since a significant effect of age on the accuracy of LAP and TyG in the overall study subjects was detected, we used an induced ROC regression for further analysis to draw more standard results (Table 3). Sex- and age-specific cut-off values are shown with their corresponding sensitivity and specificity. Figure 2 depicts the estimated cut-off values of VAI, LAP, TyG, and HOMA-IR by age in both sexes. The induced ROC regression provided evidence for a non-linear relationship between age and AUCs in the overall population (p-value for VAI: 0.945, LAP: 0.673, TyG: 0.629). Despite the evidence for a non-linear relationship between age and AUCs, fluctuations of the optimal cut-off values of VAI and LAP were observed in men and women. Specifically, while the cut-off values of VAI and LAP showed a drastic decrease at the age 70 years in men, they also showed noticeable changes between the age of 30 and 40 years rather than at 70 years in women. Unlike the VAI or LAP, TyG did not have a remarkable fluctuation of cut-off values by age, showing optimal cut-off values of 8.82 and 8.35 at age 40 years, and 8.72 and 8.48 at age 70 years, in men and women, respectively.

**Table 3:** Performance of markers for identifying persons with prediabetes/diabetes, according to sex and age.

Marker	Population		Cut-off	Sensitivity	Specificity	AUC (95% CI)
VAI	Overall	40 yrs	1.28	0.706	0.570	0.690 (0.584–0.779)
		55 yrs	1.46	0.711	0.590	0.701 (0.633–0.751)
		70 yrs	1.88	0.484	0.730	0.633 (0.525–0.719)
	Men	40 yrs	1.92	0.537	0.710	0.667 (0.572–0.770)
		55 yrs	1.10	0.868	0.340	0.639 (0.555–0.724)
		70 yrs	1.60	0.507	0.660	0.611 (0.457–0.736)
	Women	40 yrs	1.85	0.432	0.840	0.602 (0.404–0.844)
		55 yrs	1.23	0.821	0.510	0.714 (0.618–0.796)
		70 yrs	1.47	0.728	0.510	0.655 (0.530–0.765)
LAP	Overall	40 yrs	33.99	0.698	0.640	0.728 (0.642–0.821)
		55 yrs	41.78	0.720	0.640	0.743 (0.680–0.796)
		70 yrs	50.22	0.550	0.650	0.611 (0.521–0.705)
	Men	40 yrs	50.25	0.641	0.710	0.728 (0.620–0.787)
		55 yrs	63.63	0.545	0.730	0.669 (0.592–0.767)
		70 yrs	51.80	0.537	0.650	0.574 (0.458–0.739)
	Women	40 yrs	26.85	0.646	0.640	0.657 (0.492–0.849)
		55 yrs	42.98	0.650	0.740	0.746 (0.663–0.827)
		70 yrs	42.26	0.658	0.570	0.632 (0.515–0.746)
TyG	Overall	40 yrs	8.65	0.650	0.760	0.776 (0.718–0.834)
		55 yrs	8.68	0.691	0.670	0.745 (0.711–0.795)
		70 yrs	8.58	0.715	0.590	0.712 (0.625–0.779)
	Men	40 yrs	8.82	0.627	0.730	0.732 (0.647–0.800)
		55 yrs	8.80	0.666	0.630	0.689 (0.624–0.766)
		70 yrs	8.72	0.630	0.660	0.685 (0.564–0.801)
	Women	40 yrs	8.35	0.646	0.660	0.700 (0.580–0.832)
		55 yrs	8.53	0.782	0.650	0.786 (0.722–0.843)
		70 yrs	8.48	0.782	0.500	0.684 (0.590–0.781)
HOMA-IR	Overall	40 yrs	2.39	0.590	0.780	0.761 (0.665–0.861)
		55 yrs	2.31	0.686	0.770	0.810 (0.729–0.864)
		70 yrs	2.63	0.553	0.830	0.733 (0.646–0.829)
	Men	40 yrs	2.36	0.693	0.770	0.792 (0.713–0.882)
		55 yrs	2.64	0.651	0.780	0.761 (0.651–0.812)
		70 yrs	2.37	0.726	0.770	0.819 (0.714–0.915)
	Women	40 yrs	2.22	0.541	0.720	0.669 (0.593–0.907)
		55 yrs	2.26	0.833	0.800	0.892 (0.758–0.932)
		70 yrs	2.50	0.588	0.810	0.743 (0.606–0.844)

AUC (95% CI), area under the ROC curve (95% Confidence Interval). Cut-off value with corresponding sensitivity, specificity, and AUC estimated for 40, 55, and 70 years of age by induced method of non-parametric ROC regression (1000 bootstraps).

### Sensitivity analyses

There were 6.9% of men (n = 66) and 5.7% of women (n = 63) taking statins among the study participants. When these participants were excluded for the sensitivity analysis, there was no effect on the discriminatory accuracy and the optimal cut-off values of VAI, LAP, and TyG (Supplemental Table S4). Another sensitivity analysis was conducted with 1934 individuals (men: 903, women: 1031) excluding people taking statins, but including persons with a history of MI and/or stroke. This did not significantly change the diagnostic abilities of the three indices, either (Supplemental Table S5).

## Discussion

Although AUCs between 0.8–0.9 are usually regarded as good values and between 0.7–0.8 as fair values, the interpretation of AUCs of diagnostic parameters is critically dependent upon the clinical context^15^. Currently, HbA1c is used for the diagnosis of prediabetes/diabetes by ADA criteria, and its abilities to discriminate prediabetes/diabetes defined by OGTT have been demonstrated in many studies (AUC range for diabetes: 0.730–0.958, prediabetes:0.650–0.729). The present study shows that VAI, LAP, and TyG may provide discrimination of prediabetes/diabetes comparable to that of HbA1c in a European population^16–19^. Compared with HOMA-IR, all three novel markers had higher sensitivity for prediabetes/diabetes according to the maximized YI in the overall population, and the accuracy of TyG was superior to VAI and LAP. To the best of our knowledge, this is the first study that compared the three novel IR-related lipid indices (VAI, LAP, and TyG) with HOMA-IR, which is an established marker for diagnosing prediabetes/diabetes^7,20^.

The VAI was introduced for the first time by Amato *et al*.^21^ as a surrogate indicator for the definition of cardiometabolic risk in a healthy population, showing a significantly inverse correlation with insulin sensitivity. In our study population, the AUC for VAI was 0.687 (0.662–0.712), which indicates a relatively lower discriminatory ability compared to other markers. However, it was larger than the AUC from an Iranian study (0.610 [0.574–0.645]) and a Chinese study (men: 0.622 [0.607–0.636], women: 0.654 [0.642–0.665])^13,22^.

LAP, devised for the U.S. National Health and Nutrition Examination Survey, has been used as a marker of central obesity^23^. Furthermore, it is proposed as a marker indicating IR and diabetes^24^. In our analyses, the cut-off value for men was 56.7 (AUC: 0.701 [0.667–0.735]) and 30.4 (AUC: 0.760 [0.728–0.793]) for women. Several reports from Asian countries have investigated the diagnostic ability of LAP for diabetes. One Japanese study (10,170 participants aged 35–40) showed that LAP had the ideal predictive value for hyperglycemia (AUC for men: 0.764 [0.742–0.787], women: 0.763 [0.709–0.816]) and diabetes (AUC for men: 0.804 [0.767–0.840], women: 0.845 [0.757–0.933]), suggesting the same cut-off values for hyperglycemia and diabetes (men: 37.2, women: 21.1)^25^. In a Korean study (7,708 participants aged 40–69), the cut-off value of LAP for men was 30.50, and the corresponding AUC was 0.602 (0.586–0.618), while the cut-off value and the AUC of LAP for women were 35.84 and 0.623 (0.607–0.637), respectively^26^. The interesting finding from our results was that the cut-off value for male subjects was much higher than the one for female individuals, which is consistent with the Japanese study, whereas women showed a higher cut-off value compared to men in the Korean study. These differences may be related to a modified relationship between insulin resistance and body fat distribution by ethnicities^27,28^. More prospective studies on the discriminating ability of LAP for prediabetes/diabetes are needed to explain these concerns.

TyG, developed by Simental-Mendia *et al*.^11^, is well recognized as a reliable surrogate marker for identifying IR^29^. Recent studies indicate that its discriminatory ability for IR is better than that of the HOMA-IR^12^. Furthermore, several studies conducted in Asia and Europe validated the strong association between TyG and incidence of diabetes^14,26,29^. In the present study, as compared with HOMA-IR, the accuracy of TyG did not show inferiority in women (AUC: 0.790 [0.758–0.822] vs. 0.782 [0.751–0.814], p = 0.672) and individuals younger than 65 years (AUC: 0.790 [0.766–0.814] vs. 0.772 [0.747–0.797], p = 0.186). The cut-off value of TyG for men was 8.75, and the one for women was 8.53, which is consistent with previous findings. A recently conducted Korean study suggested a cut-off point of TyG for men of 8.86 (AUC: 0.623 [0.607–0.638]) and 8.52 for women (AUC: 0.644 [0.629–0.659])^14^. Other proposed cut-off values from research conducted in Spain were similar (men: 8.43, women: 8.19), and the pooled AUC was 0.750 (0.707–0.810)^30^. Regarding HOMA-IR, a previous 15-year prospective study in China suggested 1.37 (AUC: 0.753 [0.713–0.758]) as a cut-off value for discriminating prediabetes and 1.97 (AUC: 0.807 [0.777–0.886]) for diabetes, but in this case, the values were pooled in men and women^31^, and are lower than the cut-off values determined for the diagnosis of prediabetes/diabetes in the present study (men: 2.36, women: 2.57).

By using the direct method of non-parametric ROC regression, we found a lower accuracy of the three novel markers for diagnosing prediabetes/diabetes as age increases in men and women, and the influence of age was significant in the overall population analysis. Therefore, we computed each marker’s cut-off value, sensitivity, and specificity using an induced ROC regression. After applying the induced method, there was no significant linear effect of age on AUC. The results showed that the cut-off value for TyG was the most stable without fluctuation across age. Overall, the range of the variation in sensitivity, specificity, and AUC across age was wider in women than men. These differences between the sexes in our study may reflect the effect of hormonal changes by age in women, but further investigations are needed to clarify this issue.

Our study has several strengths. It is the first report comparing three novel markers for diagnosing prediabetes/diabetes in a European population. Additionally, innovative direct and induced methodologies of non-parametric ROC regression were used to analyse the influence of age on the accuracy of the markers when identifying the presence of prediabetes/diabetes. Given that VAI was modelled on a healthy population, we excluded persons with previous MI and/or stroke events in our primary analysis so as not to interfere with the diagnostic capabilities of the markers^21^. For the internal validation, a 10-fold cross-validation for the AUC estimates was carried out. We could also check the robustness of our results by using two different rigorous sensitivity analyses: (1) Analysis excluding persons taking statins; (2) Analysis excluding persons taking statins, but including individuals with a previous history of MI and/or stroke.

We also acknowledge some limitations of the present study. First, due to the cross-sectional analysis, we were not able to predict incident prediabetes/diabetes among initially healthy community-dwelling subjects. Additionally, the small sample size of younger participants might have resulted in low precision of the discriminatory accuracy in young women. Further studies including participants with an evenly distributed age range are needed to elucidate this matter. Lastly, despite a lack of change in the discriminatory capacity and cut-off values of LAP between the primary analysis and sensitivity analysis, a considerable difference in the cut-off values between male and female subjects remained without clear explanation.

In conclusion, VAI, LAP, and TyG are useful indices for identifying prediabetes/diabetes in both men and women. In particular, TyG is superior to discriminate diabetes status, showing no inferior ability compared to the HOMA-IR. One important strength of TyG is a lower cost compared to HOMA-IR, as the cost of a fasting insulin level measurement is about six times higher than the cost of a triglyceride test^32^. Although LAP indicates relatively lower accuracy than TyG and HOMA-IR in the overall population, no significant difference of AUCs between LAP and HOMA-IR in women was observed. Given its clear benefit, LAP could be a reliable parameter for diabetes screening in the female population as long as WC could be determined appropriately and at a minimum of costs in the clinical setting. Therefore, we suggest the use of LAP and TyG as accurate, convenient and cost-effective diagnostic measurements for the early detection of prediabetes/diabetes in the general population.

## Methods

### Study population

The Cooperative Health Research in the Region of Augsburg (KORA) study is a population-based study conducted in Augsburg, Germany, using the same geographical region and study methods as the former Monitoring Trends and Determinants in Cardiovascular Disease (MONICA) project of the World Health Organization (WHO). The present study data were derived from the KORA-F4 study^33^, a follow-up of the KORA-S4 survey carried out between 1999 and 2001 focusing on diabetes and cardiovascular disease. Among 4,261 subjects of the S4 baseline study, 3,080 participated in the 7-year follow-up F4 study as well. Subjects were considered ineligible for F4 if they had already died (n = 176), did not live in the study region or were lost to follow-up (n = 206), or had demanded deletion of their address data (n = 12). Of the remaining 3,867 eligible persons, 174 could not be reached, 218 were unable to show up because of illness or time constraints, and 395 did not want to be included in this follow-up examination^34^.

From the KORA-F4 dataset of 3,080 subjects, a total of 1,035 individuals were excluded because of prevalent diabetes status (type 1, 2, or medication-induced) (n = 317), history of MI and/or stroke (n = 111), or incomplete anthropometric and clinical data (n = 607). Finally, 2,045 individuals (955 men, 1,090 women; aged 31–72 years) were included in the analysis. The investigations were carried out in accordance with the Declaration of Helsinki. Written, informed consent was obtained from each study participant, and the study was approved by the ethics committee of the Bavarian Medical Association. All data generated or analysed during this study are included in this published article and its Supplementary Information file. In case the readers have questions regarding the data the corresponding author will be able to reply.

### Anthropometric and clinical assessment

Baseline information on socio-demographic variables, alcohol consumption, smoking habits, physical activity, medication use, and parental history of diabetes was collected by trained medical staff during a standardized interview. The assessment of alcohol intake was based on weekday and weekend consumption of beer, wine, and spirits. To derive the average number of grams of alcohol consumed per day (g/day), total intake was calculated by multiplying weekday consumption by five and adding this to weekend consumption, applying the following conversions: 1 liter beer = 40 g alcohol, 1 liter wine = 100 g alcohol, 1 shot distilled spirits (0.02 liter) = 6.2 g alcohol^35^. Study participants provided their smoking history (never, former, current). Individuals who participated in leisurely physical activity weekly for at least one hour in summer or winter were classified as being physically active. Also, they were requested to answer whether, and if so, which medication(s) they took within seven days before the examination.

Anthropometric measurements were taken once after removal of heavy clothing, shoes, and belts by qualified examiners who passed extensive training and a certification test. Waist and hip circumferences were measured with a measurement tape to the nearest 0.1 cm: waist midway between the lowest rib and the iliac crest; hip at the level of the gluteal protrusion. Height was measured to the nearest 0.1 cm, and body weight was measured to the nearest 0.1 kg. BMI was calculated as weight in kilograms divided by height in square meters. Systolic and diastolic blood pressure were measured three times at the right arm of seated subjects after at least 5 min at rest. The pause between readings was 3 min. The mean of the second and third measurements was calculated and used for the present analyses.

Hypertension was defined when blood pressure values were ≥140/90 mmHg and/or use of antihypertensive medication given that the persons were aware of being hypertensive^36^. Furthermore, participants went through an extensive standardized medical assessment including the collection of overnight fasting blood samples. All detailed procedures of examination are described elsewhere^37^.

### Measurement of laboratory parameters

Total cholesterol (TC) and HDL-C were measured in fresh serum by enzymatic methods (CHOL Flex and AHDL Flex, Dade Behring, Marburg, Germany). TG was measured in fresh serum with the GPO-PAP method (Dade Behring). HbA1c was quantified with a reverse-phase cation-exchange HPLC method using a Menarini–Arkray Analyser HA-8140 (Menarini Diagnostics, Florence, Italy). Serum glucose levels were assessed by the hexokinase method (GLU Flex, Dade Behring, Marburg, Germany). Serum insulin was determined by ECLIA on a cobas analyser (Roche, Mannheim, Germany).

### Definition of prediabetes and newly diagnosed diabetes

A 75 g OGTT was performed in all non-diabetic participants as described by the WHO^33^. Diabetes and prediabetes (impaired fasting glucose and/or impaired glucose tolerance) were defined according to ADA criteria published in 2015: (1) Normal: Fasting serum glucose (FSG) < 100 mg/dL (5.6 mmol/L) and 2-hour serum glucose (2h-SG) in the OGTT < 140 mg/dL (7.8 mmol/L); (2) Impaired fasting glucose (IFG): FSG 100 mg/dL (5.6 mmol/L) to 125 mg/dL (6.9 mmol/L); (3) Impaired glucose tolerance (IGT): 2-h SG in the 75-g OGTT 140 mg/dL (7.8 mmol/L) to 199 mg/dL (11.0 mmol/L); (4) Diabetes: FSG ≥ 126 mg/dL (7.0 mmol/L) or 2-h SG ≥ 200 mg/dL (11.1 mmol/L) during an OGTT^38^. According to the results of the OGTT and medical history, the study population was assigned to seven groups: normal, IFG, IGT, IFG/IGT, newly diagnosed diabetes, known type 2 diabetes, known type 1 diabetes, and known medication-induced diabetes. Since individuals with known diabetes were not comparable to the rest of subjects due to on-going drug treatment, we excluded persons with known type 2 diabetes, known type 1 diabetes, and known medication-induced diabetes. After exclusion, participants with prediabetes (IFG, IGT, IFG/IGT) and newly diagnosed diabetes were grouped together and were referred to as prediabetes/diabetes in the present study.

### Definition of VAI, LAP, TyG, and HOMA-IR

VAI and LAP were calculated using the following formula^8–10^:

$${{\rm{VAI}}}_{{\rm{men}}}=(\frac{{\rm{WC}}}{39.68+1.88\ast {\rm{BMI}}})\ast (\frac{{\rm{TG}}}{1.03})\ast (\frac{1.31}{{\rm{HDL}}-{\rm{C}}})$$

$${{\rm{VAI}}}_{{\rm{women}}}=(\frac{{\rm{WC}}}{36.58+1.89\ast {\rm{BMI}}})\ast (\frac{{\rm{TG}}}{0.81})\ast (\frac{1.52}{{\rm{HDL}}-{\rm{C}}})$$

$${{\rm{LAP}}}_{{\rm{men}}}=({\rm{WC}}-65)\ast {\rm{TG}}$$

$${{\rm{LAP}}}_{{\rm{women}}}=({\rm{WC}}-58)\ast {\rm{TG}}$$

TG was converted to mg/dl for calculation of TyG. The index of TyG and HOMA-IR were calculated^11^.

$${\rm{TyG}}=\,\mathrm{ln}(\frac{{\rm{TG}}\ast \mathrm{FSG}}{2})$$

$${\rm{HOMA}}-{\rm{IR}}=\frac{{\rm{fasting}}\,{\rm{serum}}\,{\rm{insulin}}\ast \mathrm{FSG}\,}{22.5}$$

### Statistical analyses

Continuous variables were presented as mean and standard deviation. Categorical variables were expressed as frequency (n, %). The discriminatory accuracy of the three novel markers for prediabetes and newly diagnosed diabetes was assessed by the area under the receiver operating characteristic (ROC) curve (AUC). OGTT-defined prediabetes/diabetes served as the reference. We provided sensitivity, specificity, positive predictive value (PPV), negative predictive value (NPV), and the diagnostic likelihood ratio (DLR) for each marker. The YI was used to derive optimal cut-off values^39^.

$${\rm{YI}}={\rm{sensitivity}}+{\rm{specificity}}-1$$

Due to the increased risk of diabetes among individuals who take statins, we performed sensitivity analyses excluding subjects reporting use of statins^40^. Individuals with previous history of MI and/or stroke were included in second sensitivity analysis to assess how cardio- and cerebrovascular events influence the discrimination of prediabetes/diabetes. We also conducted internal validation using a 10-fold cross-validation that computes confidence intervals for AUC estimates based on influence curves of both regular independent and identically distributed and pooled repeated measures data^41^.

Furthermore, since the discriminatory ability of each marker might vary based on individual characteristics, we used non-parametric ROC regression to examine whether each marker’s AUC is related to sex and age. Specifically, we used the non-parametric direct and induced approach proposed by Rodriguez-Alvarez *et al*.^42^. The direct method models the ROC curve using generalized additive models (ROC-GAM)^42,43^. It allowed direct evaluations of the impact of age on the ROC curve and tests for an interaction between age and sex. The induced ROC estimates the covariate-specific ROC curve in the presence of age as a one-dimensional continuous covariate. Compared to the direct method, the induced ROC approach allowed us the use of more standard regression techniques. First, we established a linear effect of age on the AUC for each marker using the direct ROC regression method. Then we conducted an additional analysis using the induced ROC regression with 1000 bootstrap replications. By doing so, the variations in the performance of markers by age could be analysed. Lastly, the bootstrap-based estimated AUC and its confidence interval were obtained. The age-specific cut-off values were computed based on the maximized YI.

The R-packages pROC, npROCRegression, OptimalCutpoints and cvAUC were used in the R software package (Version 3.5.2, R Project for Statistical Computing, Vienna, Austria). A two-tailed p-value < 0.05 was considered as statistically significant.

## Supplementary information

Supplementary info

## Data Availability

All data generated or analysed during this study are included in this published article and its supplementary information file.
