# Supplementary material for: Visceral adiposity index (VAI), lipid accumulation product (LAP), and product of triglycerides and glucose (TyG) to discriminate prediabetes and diabetes
Source: Sci Rep. 2019 Jul 4;9:9693. doi: 10.1038/s41598-019-46187-8 (PMC6609728; doi:10.1038/s41598-019-46187-8)
Supplement: Supplementary file 1 — Supplementary info [file 41598_2019_46187_MOESM1_ESM.pdf]

<Supplementary Information>

Article in *Scientific Reports*

Visceral adiposity index (VAI), lipid accumulation product (LAP), and product of triglycerides and glucose (TyG) to discriminate prediabetes and diabetes

Nayeon Ahn[1,2], Sebastian Edgar Baumeister[1], Ute Amann[1], Wolfgang Rathmann[3], Annette Peters[4], Cornelia Huth[4], Barbara Thorand[4], Christa Meisinger[1,5]

[1] Chair of Epidemiology, Ludwig-Maximilians-Universität München, UNIKA-T Augsburg, Augsburg, Germany

[2] The Institute of Medical Informatics, Biometry and Epidemiology (IBE), Ludwig-Maximilians-University of Munich, Munich, Germany

[3] Institute for Biometrics and Epidemiology, German Diabetes Center, Leibniz Center for Diabetes Research at Heinrich Heine University, Düsseldorf, Germany

[4] Institute of Epidemiology, Helmholtz Zentrum München, German Research Center for Environmental Health, Neuherberg, Germany

[5] Independent Research Group Clinical Epidemiology, Helmholtz Zentrum München, German Research Center for Environmental Health, Neuherberg, Germany

Supplemental table S1. General Distribution of cut-off value with corresponding predictive values of VAI, LAP, and TyG

	Sensitivity	Specificity	PPV	NPV	AUC (95% CI)	P-value	DLR(+)	DLR(-)	YI	Cut-offs
**Overall**										
VAI	0.645	0.629	0.432	0.802	0.687 (0.662-0.712)	<0.001 †	1.738	0.565	0.274	1.52
LAP	0.698	0.663	0.475	0.834	0.743 (0.720-0.765)	<0.01 ‡	2.073	0.456	0.361	41.30
TyG	0.732	0.658	0.484	0.849	0.762 (0.740-0.784)	<0.001 §	2.142	0.408	0.390	8.60
**Men**										
VAI	0.433	0.792	0.562	0.693	0.649 (0.613-0.685)	<0.001 †	2.076	0.716	0.224	2.33
LAP	0.595	0.727	0.574	0.744	0.701 (0.667-0.735)	0.025 ‡	2.179	0.558	0.322	56.70
TyG	0.666	0.663	0.550	0.762	0.721 (0.687-0.754)	<0.001 §	1.974	0.504	0.328	8.75
**Women**										
VAI	0.755	0.582	0.358	0.885	0.720 (0.685-0.755)	<0.001 †	1.807	0.421	0.337	1.32
LAP	0.802	0.613	0.390	0.909	0.760 (0.728-0.793)	0.047 ‡	2.074	0.323	0.415	30.40
TyG	0.720	0.705	0.429	0.891	0.782 (0.751-0.814)	<0.001 §	2.438	0.398	0.425	8.53
**Age≥ 65**										
VAI	0.525	0.702	0.676	0.555	0.639 (0.580-0.699)	0.142 †	1.762	0.676	0.227	1.87
LAP	0.592	0.675	0.684	0.583	0.665 (0.607-0.723)	0.052 ‡	1.825	0.604	0.268	49.95
TyG	0.508	0.781	0.734	0.573	0.697 (0.641-0.753)	<0.001 §	2.326	0.629	0.290	8.85
**Age<65**										
VAI	0.650	0.637	0.384	0.839	0.695 (0.667-0.723)	<0.001 †	1.790	0.549	0.287	1.52
LAP	0.695	0.682	0.432	0.865	0.750 (0.724-0.776)	<0.01 ‡	2.184	0.447	0.377	41.30
TyG	0.752	0.671	0.443	0.886	0.772 (0.747-0.797)	<0.001 §	2.287	0.370	0.423	8.60

DLR, Diagnostic Likelihood Ratio; YI, Youden Index; PPV, positive predictive value; NPV, negative predictive value
† P-value of ROC test between the area under the curve (AUC) value for visceral adiposity index (VAI) and that of lipid accumulation product (LAP).
‡ P-value of ROC test between the area under the curve (AUC) value for LAP and that of the product of triglycerides and glucose (TyG).
§ P-value of ROC test between the area under the curve (AUC) value for TyG and VAI.

Supplemental table S2. Performance of markers for identifying persons with prediabetes/diabetes, according to sex and age

Marker	Population		ROC coefficient	P- value	AUC (95% CI)*
VAI		40yrs			0.702 (0.654-0.745)
	Overall	55yrs	-0.0089	0.0725	0.667 (0.641-0.695)
		70yrs			0.631 (0.591-0.678)
		40yrs			0.666 (0.611-0.721)
	Men	55yrs	-0.0066	0.3025	0.640 (0.604-0.677)
		70yrs			0.613 (0.540-0.676)
		40ysr			0.707 (0.629-0.778)
	Women	55yrs	-0.0069	0.3750	0.681 (0.638-0.721)
		70yrs			0.654 (0.585-0.720)
LAP		40yrs			0.747 (0.702-0.786)
	Overall	55yrs	-0.0107	0.0250	0.709 (0.684-0.734)
		70yrs			0.668 (0.623-0.713)
		40yrs			0.699 (0.642-0.755)
	Men	55yrs	-0.0060	0.3425	0.675 (0.639-0.713)
		70yrs			0.651 (0.581-0.715)
		40yrs			0.728 (0.651-0.800)
	Women	55yrs	-0.0065	0.4100	0.704 (0.664-0.746)
		70yrs			0.679 (0.613-0.741)
TyG		40yrs			0.769 (0.727-0.808)
	Overall	55yrs	-0.0110	0.0250	0.732 (0.709-0.755)
		70yrs			0.692 (0.650-0.734)
		40yrs			0.725 (0.670-0.775)
	Men	55yrs	-0.0059	0.3750	0.703 (0.668-0.738)
		70yrs			0.680 (0.607-0.740)
		40yrs			0.762 (0.689-0.832)
	Women	55yrs	-0.0100	0.1900	0.729 (0.688-0.768)
		70yrs			0.693 (0.630-0.754)
HOMA-IR		40yrs			0.777 (0.735-0.818)
	Overall	55yrs	-0.0004	0.9475	0.776 (0.754-0.799)
		70yrs			0.775 (0.735-0.813)
		40yrs			0.770 (0.718-0.819)
	Men	55yrs	-0.0020	0.7525	0.764 (0.730-0.796)
		70yrs			0.757 (0.696-0.812)
		40yrs			0.754 (0.675-0.823)
	Women	55yrs	0.0051	0.5675	0.772 (0.736-0.808)
		70yrs			0.790 (0.725-0.845)

*AUC (95% CI), area under the ROC curve (95% Confidence Interval). A direct methodology ROC regression models incorporating age as covariate.

Supplemental figure S3. Influence of sex and age on the area under the ROC curve (AUC) by direct non-parametric ROC regression models.

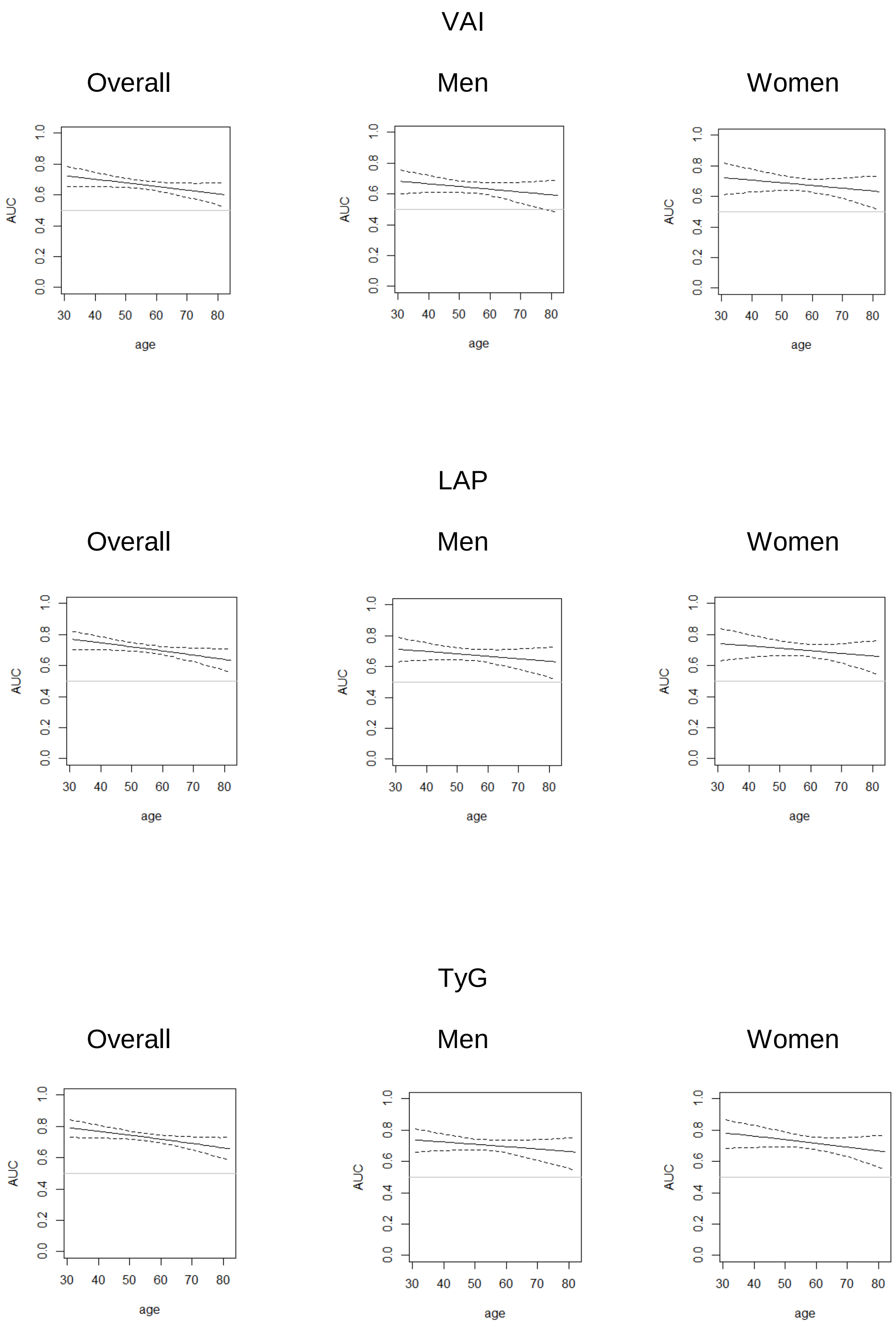

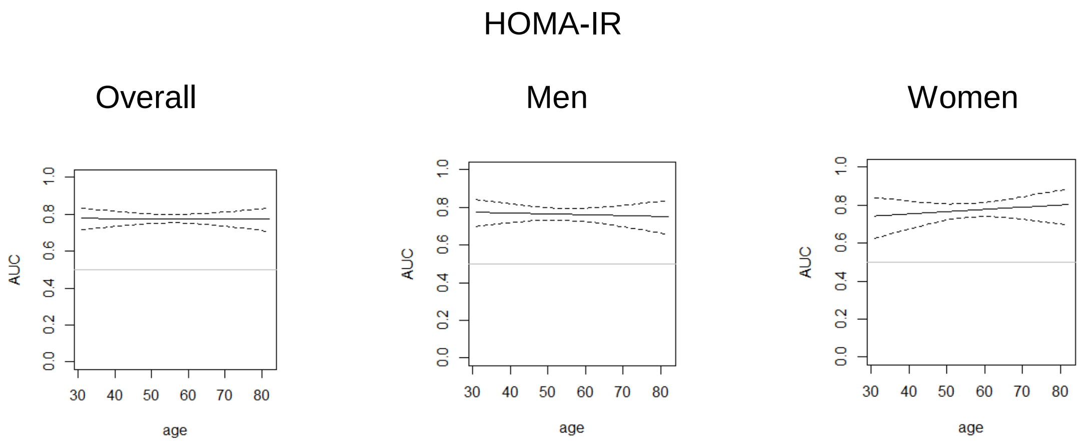
HOMA-IR
Overall
Men
Women
AUC
age
0.0
0.2
0.4
0.6
0.8
1.0
30
40
50
60
70
80

Supplemental table S4. Sensitivity analysis (1) of the discriminatory accuracy and cut-off values for VAI, LAP, TyG, and HOMA-IR

	Sensitivity	Specificity	PPV	NPV	AUC (95% CI)	YI	Cut-offs	P-value†
**Overall**								
VAI	0.675	0.604	0.415	0.817	0.691 (0.665-0.716)	0.279	1.44	0.853
LAP	0.694	0.673	0.469	0.841	0.746 (0.723-0.770)	0.368	41.30	0.821
TyG	0.734	0.669	0.480	0.858	0.765 (0.742-0.788)	0.402	8.60	0.861
HOMA-IR	0.631	0.796	0.563	0.838	0.789 (0.767-0.812)	0.427	2.37	0.945
**Men**								
VAI	0.434	0.801	0.565	0.703	0.658 (0.621-0.695)	0.234	2.33	0.190
LAP	0.596	0.734	0.572	0.753	0.706 (0.671-0.741)	0.331	56.70	0.096
TyG	0.657	0.689	0.558	0.771	0.727 (0.693-0.761)	0.346	8.78	0.065
HOMA-IR	0.687	0.722	0.595	0.794	0.779 (0.748-0.810)	0.408	2.27	0.652
**Women**								
VAI	0.736	0.598	0.347	0.886	0.715 (0.678-0.753)	0.334	1.34	0.354
LAP	0.805	0.608	0.373	0.915	0.760 (0.726-0.794)	0.413	29.60	0.667
TyG	0.714	0.711	0.418	0.896	0.780 (0.747-0.813)	0.425	8.53	0.451
HOMA-IR	0.571	0.861	0.543	0.874	0.786 (0.752-0.820)	0.432	2.57	0.988
**Age>=65**								
VAI	0.527	0.711	0.693	0.548	0.643 (0.578-0.709)	0.237	1.87	0.283
LAP	0.613	0.678	0.702	0.586	0.669 (0.605-0.733)	0.291	49.95	0.359
TyG	0.513	0.802	0.762	0.571	0.697 (0.635-0.758)	0.315	8.85	0.448
HOMA-IR	0.633	0.860	0.848	0.654	0.793 (0.740-0.847)	0.493	2.65	0.965
**Age<65**								
VAI	0.644	0.644	0.377	0.844	0.696 (0.667-0.725)	0.288	1.52	0.852
LAP	0.683	0.691	0.425	0.867	0.750 (0.723-0.776)	0.374	41.30	0.807
TyG	0.751	0.751	0.442	0.891	0.773 (0.747-0.799)	0.432	8.60	0.967
HOMA-IR	0.642	0.766	0.479	0.864	0.783 (0.758-0.809)	0.408	2.27	0.905

Sensitivity analysis excluding individuals taking statins (n, (%)); Men: 66 (6.9%), Women: 63 (5.7%)

VAI, visceral adiposity index; LAP, lipid accumulation product; TyG, triglyceride and glucose PPV, positive predictive value; NPV, negative predictive value; YI, Youden Index

† P-value of receiver operating characteristic (ROC) test between AUC of each marker from original dataset and sensitivity analysis dataset, respectively.

Supplemental table S5. Sensitivity analysis (2) of the discriminatory accuracy and cut-off values for VAI, LAP, TyG, and HOMA-IR

	Sensitivity	Specificity	PPV	NPV	AUC (95% CI)	YI	Cut-offs	P-value†
**Overall**								
VAI	0.678	0.604	0.419	0.816	0.690 (0.664-0.716)	0.281	1.44	0.970
LAP	0.697	0.671	0.472	0.840	0.746 (0.722-0.769)	0.367	41.30	0.964
TyG	0.735	0.668	0.483	0.857	0.765 (0.742-0.788)	0.404	8.60	0.995
HOMA-IR	0.662	0.765	0.544	0.843	0.789 (0.767-0.811)	0.427	2.27	0.999
**Men**								
VAI	0.433	0.800	0.569	0.698	0.657 (0.620-0.693)	0.233	2.33	0.522
LAP	0.594	0.733	0.575	0.747	0.704 (0.670-0.739)	0.326	56.70	0.600
TyG	0.661	0.688	0.564	0.769	0.726 (0.692-0.760)	0.349	8.78	0.579
HOMA-IR	0.681	0.731	0.607	0.790	0.779 (0.749-0.810)	0.412	2.29	0.928
**Women**								
VAI	0.737	0.597	0.347	0.887	0.716 (0.679-0.753)	0.334	1.34	0.510
LAP	0.806	0.606	0.373	0.915	0.760 (0.726-0.794)	0.412	29.60	0.595
TyG	0.716	0.708	0.416	0.896	0.780 (0.747-0.813)	0.424	8.53	0.619
HOMA-IR	0.573	0.859	0.541	0.874	0.786 (0.752-0.819)	0.432	2.57	0.957
**Age>=65**								
VAI	0.535	0.715	0.703	0.550	0.648 (0.584-0.713)	0.251	1.87	0.137
LAP	0.619	0.683	0.711	0.587	0.673 (0.610-0.736)	0.302	49.95	0.695
TyG	0.523	0.805	0.771	0.572	0.703 (0.642-0.763)	0.327	8.85	0.867
HOMA-IR	0.716	0.764	0.793	0.681	0.785 (0.731-0.839)	0.480	2.25	0.647
**Age<65**								
VAI	0.644	0.643	0.380	0.842	0.694 (0.664-0.723)	0.288	1.,52	0.495
LAP	0.683	0.689	0.426	0.865	0.748 (0.721-0.774)	0.371	41.30	0.817
TyG	0.749	0.681	0.443	0.889	0.772 (0.746-0.797)	0.430	860	0.873
HOMA-IR	0.582	0.829	0.535	0.854	0.784 (0.759-0.809)	0.411	2.53	0.828

Sensitivity analysis excluding individuals taking statins, but including individuals with previous MI and/or stroke (Overall: 1934, Men: 903, Women:1031)
VAI, visceral adiposity index; LAP, lipid accumulation product; TyG, triglyceride and glucose PPV, positive predictive value; NPV, negative predictive value; YI, Youden Index
† P-value of receiver operating characteristic (ROC) test between AUC of each marker from original dataset and sensitivity analysis dataset, respectively.
